# Carbon foot printing school meals: data linkage and engagement activity

**DOI:** 10.23889/ijpds.v8i3.2294

**Published:** 2023-09-11

**Authors:** Alexandra Dalton, Emily Ennis, Melinda Green, Michelle A Morris

**Affiliations:** 1 Consumer Data Research Centre, University of Leeds; 2 School of Food Science and Nutrition, University of Leeds

## Introduction & Background

Food production is a substantial contributor to greenhouse gas emissions and climate change. A more sustainable diet is often a healthier one, so making lower carbon food choices serves to benefit the planet and person. In order to understand the carbon footprint of food choices, linkage of recipe information to carbon footprint data and transaction records is required. To inform positive change insights from data linkage must be communicated to the target audience, in this case schoolchildren.

## Objectives & Approach

School dinner recipe information and transaction records for school meals at five schools for a six week period were acquired. Carbon footprint estimates were calculated for each recipe, using published data. An automated dashboard was created in order that these calculations could be replicated by catering teams. Carbon footprints were appended to the school transaction records for meal choices. An interactive web game was created in ‘top trump’s' style using a selection of the recipes, with carbon footprint and popularity ranking, generated from the transaction records.

## Relevance to Digital Footprints

Transactional meal sales data from schools are digital footprint data. In this work we link these digital footprint data to detailed recipe information with estimated carbon footprints from an open data source.

## Results

The Consumer Data Research Centre Carbon Calculator and The Planet Plates game were created. The Carbon Calculator is being used in a number of settings to support food procurement and recipe development. The Planet Plates game has been used in Leeds Schools to empower schoolchildren to make positive changes to lower the carbon footprint of their meal choices. The children were engaged with all the activities and not only learned about sustainability of their food choices, but about how data they generate can be used anonymously for public good.

## Conclusions & Implications

Data linkage of digital footprint data is a powerful tool for behaviour change to tackle some of the world’s most pressing challenges. Methods and insights should be shared widely and made accessible to a range of stakeholders wherever possible.

